# Rediscovering the genus *Lyticum*, multiflagellated symbionts of the order *Rickettsiales*

**DOI:** 10.1038/srep03305

**Published:** 2013-11-22

**Authors:** Vittorio Boscaro, Martina Schrallhammer, Konstantin A. Benken, Sascha Krenek, Franziska Szokoli, Thomas U. Berendonk, Michael Schweikert, Franco Verni, Elena V. Sabaneyeva, Giulio Petroni

**Affiliations:** 1 Biology Department, University of Pisa; 2 Institute of Hydrobiology, Technische Universität Dresden; 3 Department of Cytology and Histology, St.-Petersburg State University; 4 Department of Zoology, Biological Institute, Stuttgart University; 5 These authors contributed equally to this work.

## Abstract

Among the bacterial symbionts harbored by the model organism *Paramecium*, many still lack a recent investigation that includes a molecular characterization. The genus *Lyticum* consists of two species of large-sized bacteria displaying numerous flagella, despite their inability to move inside their hosts' cytoplasm. We present a multidisciplinary redescription of both species, using the deposited type strains as well as newly collected material. On the basis of 16S rRNA gene sequences, we assigned *Lyticum* to the order *Rickettsiales*, that is intensely studied because of its pathogenic representatives and its position as the extant group most closely related to the mitochondrial ancestor. We provide conclusive proofs that at least some *Rickettsiales* possess actual flagella, a feature that has been recently predicted from genomic data but never confirmed. We give support to the hypothesis that the mitochondrial ancestor could have been flagellated, and provide the basis for further studies on these ciliate endosymbionts.

In the mid-twentieth century, T. Sonneborn revealed two features of *Paramecium* that had a lasting impact beyond the field of protozoology. First, he identified many morphologically identical strains of “*Paramecium aurelia*” that were not sexually compatible^1^. This observation provided one of the first and most extreme cases of a “sibling species” complex – fifteen different species have been described within the *P. aurelia* species complex until now^2^,^3^. Another important discovery was that of “cytoplasmic particles” of various kinds, found many times in several *Paramecium* species and often able to confer non-genetically inherited traits^4^. Years later, all these particles were identified as bacterial endosymbionts^5^.

Many of these symbionts have peculiar biological properties, and sometimes remarkably distinctive morphologies. Examples include the infectious *Holospora* with its specialized nucleus-invading form^6^, and the “killer” symbionts, that confer to infected paramecia the ability to kill uninfected “sensitive” strains present in the same culture medium^5^,^7^. Much interest was directed to the unusual bacteria belonging to genus *Caedibacter* (formerly “kappa particles”) and their complex cytoplasmic inclusions, the “R-bodies”^8^,^9^,^10^,^11^,^12^,^13^. Other equally intriguing killer symbionts were characterized, and among them were those belonging to the genus *Lyticum*^4^,^5^.

*Lyticum* bacteria appear as large rods (2.0–10.0 μm long) harbored in the hundreds in the cytoplasm of three different species of the *P.* *aurelia* complex^4^,^5^,^14^,^15^. They are non-motile, despite being covered by numerous flagella^16^. The two species were formally described as *Lyticum flagellatum* (formerly “lambda particle”, type species of the genus) and *Lyticum sinuosum* (“sigma particle”)^17^. They differ in shape (respectively, straight *vs.* curved rods) and host specificity (respectively, *Paramecium tetraurelia* or *Paramecium octaurelia* *vs.* *Paramecium biaurelia*)^5^,^18^.

The original descriptions of *Lyticum* and many other symbionts detected in the last century left many questions unanswered. One of the most important issues from an evolutionary point of view concerns the phylogenetic relationships of these bacteria.

The study of prokaryotic symbionts of protozoa is currently attracting a renewed interest, and is performed with the aid of molecular tools complementing ultrastructural methods like electron microscopy [e.g.^19^,^20^,^21^,^22^,^23^,^24^,^25^]. In recent years, the focus has shifted to the remarkable biodiversity of these organisms and their close relationships with human pathogens, e.g. *Rickettsia*^26^,^27^,^28^,^29^ and *Francisella*^30^,^31^.

In this work, we have characterized the symbionts of *P. octaurelia* strain 299 and *P. biaurelia* strain 114 following a multidisciplinary approach. They represent the type strains of *L. flagellatum* and *L. sinuosum*, respectively. We also reported a recently sampled environmental isolate (*P. biaurelia* USBL-36I1) infected by *L. sinuosum*, for which only one host strain was known so far. Morphology, ultrastructure and killer capabilities of the two bacterial species were investigated and molecular tools for their identification developed and tested. Moreover, we established their phylogenetic relationships, placing them inside the order *Rickettsiales* (*Alphaproteobacteria*) together with other obligate intracellular symbionts. This discovery not only clarifies the *Lyticum* affiliation, but also provides evidence supporting the hypothesis that *Rickettsiales*, the extant bacteria most closely related to the mitochondrial ancestor^32^,^33^,^34^,^35^, were ancestrally flagellated^36^. This finding provides a relevant contribution in inferring the features of the free-living ancestor of both *Rickettsiales* and mitochondria, supporting the view that it was motile.

## Results

### Morphology and ultrastructure

**Figure 1:**
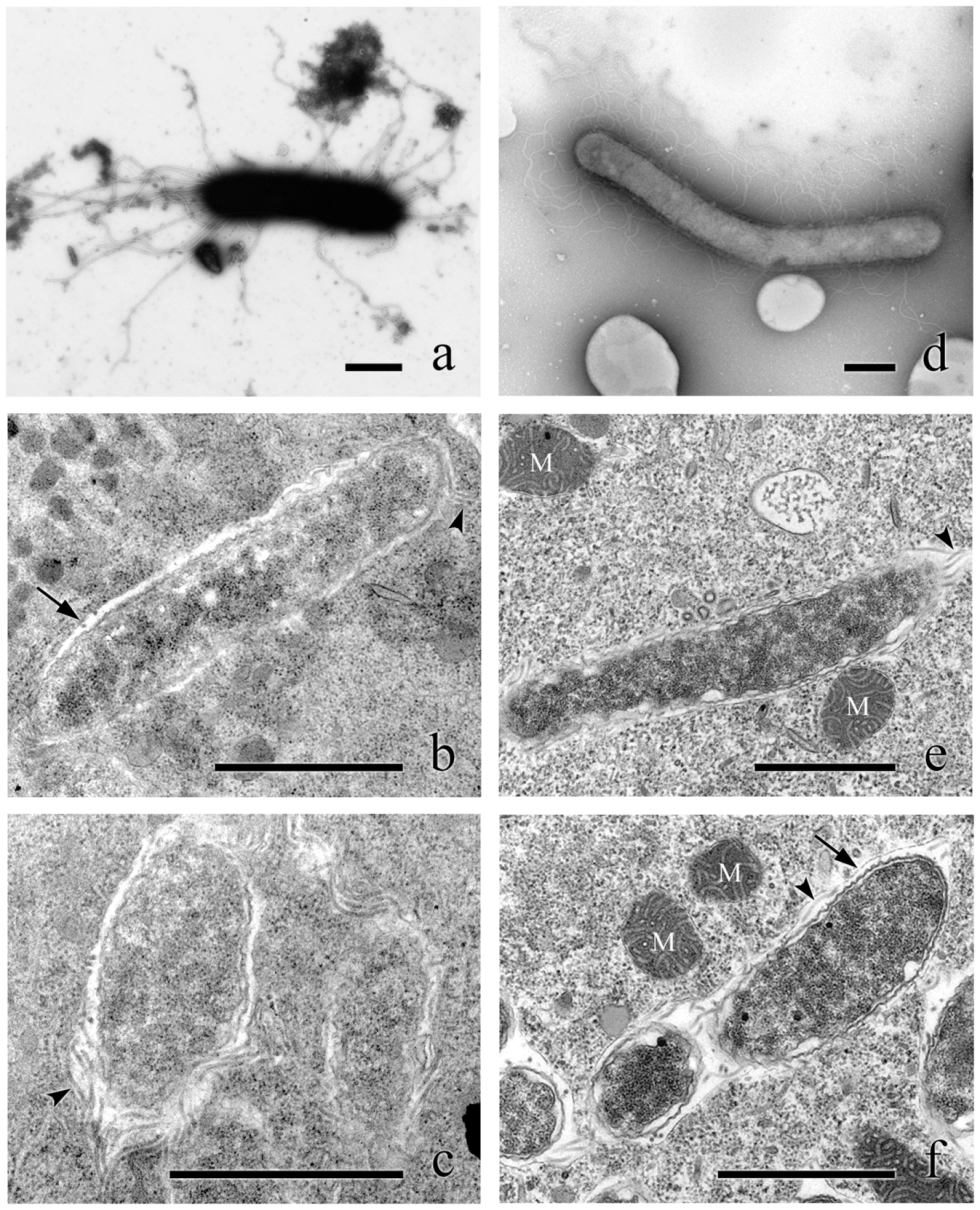
Morphology and ultrastructure of *Lyticum* species. Negative staining (a) and ultrathin sections (b, c) of *L. flagellatum* harbored by *P. octaurelia* strain 299. Negative staining (d) and ultrathin sections (e, f) of *L. sinuosum* harbored by *P. biaurelia* strain USBL-36I1. Bars stand for 1 μm. Arrowheads highlight some of the flagella, arrows point at symbiosomal membranes. M, mitochondria.

The cytoplasmic symbionts of *P. octaurelia* 299 (*L. flagellatum*) are straight rod-shaped bacteria 0.6–0.9 × 2.0–4.0 μm in size (Fig. 1a–c), while those harbored by *P. biaurelia* USBL-36I1 are bigger – up to 1.1 × 7.8 μm – and curved (Fig. 1d–f), perfectly fitting the description of *L. sinuosum*. Both are covered by numerous thick, peritrichous flagella about 4 μm long, clearly visible in TEM sections and negative staining. Nevertheless, *in vivo* observations did not show any sign of motility. The cytoplasm of both kinds of bacteria is homogeneous, with no visible inclusion. They both feature a Gram-negative type cell organization, with two membranes, and the symbionts are enclosed in a membrane-bound vesicle, often with several bacteria inside the same vesicle (Fig. 1b, f). These results are in good accordance with previous descriptions^5^.

### Molecular characterization

The 16S rRNA gene sequences of the symbionts harbored by strains 114 and USBL-36I1 are identical. They differ by 6 out of 1331 (0.5%) sites from the homologous sequence of the 299 symbiont. The most similar sequences available according to NCBI blastn are those of the *Acanthamoeba* spp. UWC8 and UWC36 symbionts (87.1–88.0% similarity), which belong to the “*Candidatus* Midichloriaceae” family within *Rickettsiales*^37^.

**Figure 2:**
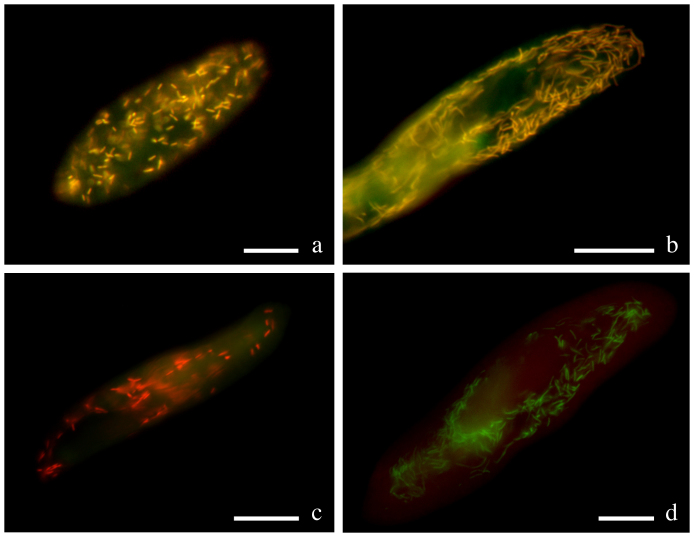
Genus- and species-specific *in situ* detection of *Lyticum flagellatum* and *Lyticum sinuosum*. Merge of the signals from probes EUB338 marked with fluorescein (green) and LytiProb_433 marked with Cy3 (red) on *P. octaurelia* strain 299 (a) and *P. biaurelia* strain USBL-36I1 (b). The signals coincide, and *Lyticum* bacteria appear yellowish. Merge of the signals from probes Lflag_268 marked with Cy3 (red) and Lsinu_268 marked with fluorescein (green) on *P. octaurelia* strain 299 (c) and *P. biaurelia* strain USBL-36I1 (d). At 20% formamide concentration, the probes used in competition are able to discriminate between the species. Bars stand for 20 μm.

Hybridizations with the genus-specific oligonucleotide probe LytiProb_433 (that provides no match on RDP) gave clear signals deriving from bacteria localized in both 299 and USBL-36I1 cells at formamide concentrations in the range of 0–50% (with an optimum at 30%). *Lyticum* bacteria were always present in all examined paramecia, usually numbering in the hundreds, but sometimes far fewer – especially in the case of 299. *L. flagellatum* bacteria in 299 were often found concatenated in groups of 2 or more cells. Double hybridizations with the eubacterial probe EUB338 demonstrated that there are no other intracellular bacteria hosted by the *Paramecium* strains studied (Fig. 2a, b). The species-specific probes Lflag_268 (providing only 2 matches on RDP, both corresponding to uncultured *Proteobacteria*) and Lsinu_268 (providing 546 matches on RDP, but only 5 inside the order *Rickettsiales*) used in competition were able to discriminate between 299 and USBL-36I1 symbionts at formamide concentrations in the range of 10–20% (with an optimum at 20%; Fig. 2c, d).

### Molecular phylogeny

**Figure 3:**
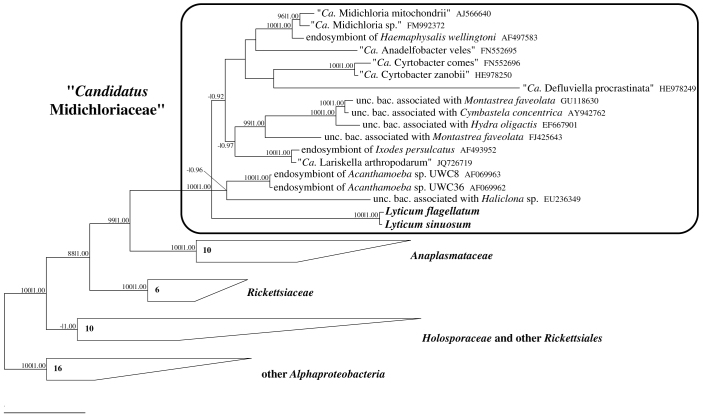
Phylogenetic position of *Lyticum* species. Bayesian tree built on the unmodified character matrix (60 sequences, 1331 characters) employing the GTR + I + G model (with the continuous gamma distribution approximated by 4 discrete categories). Numbers associated to each node correspond to ML bootstrap values and posterior probability values (values below 70|0.85 are omitted); numbers inside trapezoids show the number of sequences used to represent that clade. The bar stands for an estimated sequence divergence of 10%. *Ca.*, *Candidatus*; unc. bac., uncultured bacterium.

Details of tree topology differ, according to the method and the character matrix employed, especially within the families “*Ca.* Midichloriaceae” and *Holosporaceae* *sensu lato*^18^. Nevertheless, all trees recover the monophyly of the *Rickettsiales* families, including *Holosporaceae* *sensu stricto*^18^,^25^, and their relative positions, confirming other recent 16S rRNA analyses^37^,^38^. All trees confirm the monophyly of the *Lyticum* genus, as suggested by high similarity values between the strains, and its association to the candidate family *Midichloriaceae* within *Rickettsiales* (Fig. 3). The exact relationships within this family are not clearly resolved; however, the four genera of ciliate symbionts affiliated to this clade (“*Ca.* Anadelfobacter”, “*Ca.* Cyrtobacter”, “*Ca.* Defluviella” and *Lyticum*) do not form a monophyletic group.

### Killer effect

No killer effect was detected in any of the performed experiments. The number of living cells did not decrease in the treatments nor in the controls, and the pre-lethal symptoms described by Jurand and colleagues^39^ were never observed.

## Discussion

The infected *Paramecium* strains 299 and 114 were sampled almost a century ago^4^. Nevertheless, cultures of these ciliates still retain their original symbionts, although those of strain 114 are almost instantly lost after adaptation to standard cultivation conditions. On the other hand, the stability of the *L. flagellatum*-*P. octaurelia* 299 relationship supports the hypothesis that the symbiosis is obligate for the host, which possibly depends on metabolites provided by the bacterium^40^.

*L. sinuosum* has been reported so far only in *P. biaurelia* 114. We obtained a new environmental isolate of *P. biaurelia* which is infected by the same bacterial species, as can be inferred by morphology and the identity of 16S rRNA gene sequences. Interestingly, the monoclonal strain *P. biaurelia* USBL-36I1 was established from a water sample collected in the surroundings of the Indiana University, where T. Sonneborn was working at the time of his *Lyticum* description.

The morphological difference between the two *Lyticum* species corresponds to a difference in their 16S rRNA gene sequences, albeit small. Due to the diagnostic characters separating the two bacteria and the species-specific probes herein developed we recommend maintaining their status of separate but closely related species.

Although the identification of the described symbionts is sound, we could not repeat previous results concerning the killer trait. This was not entirely unexpected: the original literature describes the death of non-infected paramecia induced by *Lyticum* as extremely rapid (10–40 minutes), but triggered only in some *Paramecium* strains belonging to *P. triaurelia*, *P. pentaurelia* and *P. novaurelia*. Those strains were not available for the killer tests performed in this study. Therefore, our results suggest that those sensitive strains were the exception, and not the rule. The common adaptive explanation of the killer trait as a competitive advantage for the hosts^11^,^41^ would not apply to *Lyticum*, which apparently has no effect on most strains of the *P.* *aurelia* complex, including those belonging to the same species as their hosts (*P. biaurelia*, *P. tetraurelia* and *P. octaurelia*). It is also possible, of course, that the *Lyticum* killer effect requires specific physiological conditions in the sensitive, the killer and/or its symbiotic bacteria, and that those requirements were not met in our experiments. However, also the recently sampled strain USBL-36I1 did not act as a killer. This result makes it highly unlikely that an “ageing” effect of the cultures is responsible for the loss of killer activity.

*Lyticum* clearly belongs to the recently established candidate family “*Ca.* Midichloriaceae”^37^ within *Rickettsiales*, like several other symbionts of ciliates^24^,^38^, amoebas^42^ and metazoa [e.g.^43^,^44^]; a member of this group was also associated to fish disease^45^. The present study enables, for the first time, the assignment of a valid genus to this clade. Like other cytoplasmic bacteria belonging to “*Ca.* Midichloriaceae”^24^,^44^ and *Anaplasmataceae*^46^, but in contrast to members of *Rickettsiaceae*^26^,^27^,^46^, *Lyticum* symbionts are enclosed with an additional membrane, likely of host origin.

On the basis of genome annotations and phylogenomic analyses recently performed on “*Candidatus* Midichloria mitochondrii”, a hypothesis concerning the presence of flagella and motility in the *Rickettsiales*-mitochondria ancestor was proposed^36^, even though none of the so far characterized *Rickettsiales* bacteria actually possesses flagellar structures. Additionally to genome-derived evidences, further support is provided by the expression of flagellar genes on RNA and in one case also on protein level by “*Ca.* Midichloria mitochondrii”^47^. This hypothesis would confer an important role to motility in the establishment of the ancient symbiotic relationship that turned free-living bacteria into organelles. Our results support this view, revealing for the first time that heavily flagellated bacteria can be found among members of the order, and suggesting that the last common ancestor of *Rickettsiales*, or at least of “*Ca.* Midichloriaceae”, possessed flagella. The next step required for corroborating this scenario would be obtaining the sequence of *Lyticum* flagellar genes, and comparing them with those found in the “*Ca.* Midichloria mitochondrii” genome to test the alternative hypothesis that they were acquired independently.

“*Ca.* Midichloria mitochondrii” displays no flagella and is non-motile. Curiously enough, the *Lyticum* species do not use their flagella for movement. The question arises whether flagella or single flagellar proteins can also serve other than locomotion related functions. In a syntrophic symbiosis between a fermentative bacterium and a methanogenic archaeon, the significant role of the flagellar cap protein FliD to synchronize their metabolism was described^48^. One might speculate about an involvement of the numerous *Lyticum* flagella in establishment or maintenance of the symbiosis with *Paramecium*, hence this question awaits future analyses.

## Methods

### Hosts identification and culture

The *P. octaurelia* strain 299 and the *P. biaurelia* strain 114 were kindly provided by T. G. Doak and M. Lynch (Indiana University). The *P. biaurelia* strain USBL-36I1 was collected in 2011 from a small pond near Spencer (IN, USA, 39°17′45″N, 86°48′1″W). In order to confirm the identity of the host strains, morphological diagnostic features were checked^49^ and the mitochondrial cytochrome *c* oxidase subunit 1 gene (*cox1*) was sequenced according to Barth and colleagues^50^; sequences are available at EMBL database with the accession numbers HF969031-3. The cultures were maintained at 19°C on a 12:12 h light/dark cycle and fed with *Raoultella planticola* inoculated in modified Cerophyl medium according to Boscaro and colleagues^25^ or, alternatively, with *Enterobacter aerogenes* inoculated in lettuce medium at room temperature. Strain 114 was obtained several times, but the symbionts were always lost shortly after the paramecia started to propagate. Thus, Transmission Electron Microscopy (TEM), fluorescence *in situ* hybridizations (FISH) and killer tests could not be performed on this strain.

### Transmission electron microscopy

Ciliate cells were harvested by gentle centrifugation and fixed with 2.5% glutardialdehyde in 0.1 M Cacodylate buffer (pH 7.4) for 1 hour at room temperature. After washing in buffer, cells were post-fixed in 1% OsO_4_ in 0.1 M Cacodylate buffer (1 hour at room temperature). Three washing steps in this buffer were performed prior to dehydration in an acetone series and consecutive infiltration into Spurrs resin^51^. After ultrathin sectioning, sections were post-stained with 1% aqueous uranyl acetate and lead citrate^52^. Images were taken with a Zeiss EM 10 electron microscope at 60 kV. Alternatively, the cells were fixed in a mixture containing 2.5% glutaraldehyde and 1.6% paraformaldehyde in 0.1 M phosphate buffer (pH 7.4) for 2 hours at room temperature followed by a wash in the same buffer containing 12.5% sucrose and post-fixation in 1.6% OsO_4_ (1 hour at 4°C). The cells were dehydrated through a graded series of alcohol and acetone and embedded in Epoxy embedding medium (Fluka, BioChemika). Polymerization was carried out according to the manufacturer's protocol. Ultrathin sections were cut using a Reichert-Jung Ultracut E or Leica UC6, and stained with aqueous 1% uranyl acetate and 1% lead citrate. The samples were visualized using a Jeol JEM-1400 at 89 kV.

For negative staining of bacteria, several *Paramecium* cells were briefly washed in distilled water, squashed with a thin glass capillary in a drop of water, and a drop of the resulting suspension was placed on a Pioloform coated grid. Bacteria were allowed to precipitate for 2–3 min, then a drop of 1% uranyl acetate in distilled water was added for no longer than 1 min. The liquid was then absorbed with filter paper and the grid was air-dried.

### 16S rRNA gene sequencing

The almost complete 16S rRNA gene sequences were obtained through several PCR amplifications of overlapping regions and direct sequencing of the products (299 symbiont), or through cloning of PCR products, RFLP analyses and sequencing of 5 clones showing the most represented pattern to produce a consensus (for details of primers and PCR reactions, see Supplementary Methods online). The sequences are available at EMBL with the accession numbers HF969034-44.

### FISH

Hybridizations were performed according to the protocol of Manz and colleagues^53^ on individually collected *Paramecium* cells fixed with 2% paraformaldehyde (w/v). Preliminary FISH experiments were performed with the eubacterial probe EUB338^54^ and the alphaproteobacterial probe ALF1b^53^. Oligonucleotide probes specific for the obtained 16S rRNA gene sequences were developed [LytiProb_433 5′-TATCTTCCCCACCAAAAGAAC-3′, genus *Lyticum* specific; Lflag_268 5′-GCTAAAGATCGAAGCCTTGGTAA-3′, *L. flagellatum* specific; Lsinu_268 5′-GCTAAAGATCGTAGCCTTGGTAA-3′, *L. sinuosum* specific]. These novel probes were tested with a wide range (0–50%) of formamide concentrations in the hybridization buffer. *Paramecium* strains containing different alphaproteobacterial symbionts were employed as negative controls. Probe specificities were checked also *in silico* with the ProbeMatch tool of the Ribosomal Database Project (RDP) website^55^ and probe data were deposited at probeBase^56^.

### Phylogenetic analyses

Non-identical 16S rRNA gene sequences obtained were aligned with 42 homologous sequences of *Rickettsiales* bacteria and 16 of non-*Rickettsiales* alphaproteobacteria (as outgroup) using the ARB software package^57^. Sequence lengths were reduced to that of the shortest one, then multiple character matrices were produced according to Boscaro and colleagues^38^; unless otherwise stated, similarity values were calculated on the unmodified dataset. Maximum likelihood (ML) phylogenetic analyses were performed with PhyML^58^, employing bootstrap analysis (1,000 pseudoreplicates) to evaluate the reliability of nodes. Bayesian inference (BI) analyses were performed with MrBayes^59^, using three different runs with three heated and one cold chain each, iterating for 1,000,000 generations. The evolutionary model was selected according to the Akaike information criterion calculated by jModelTest^60^.

### Killer tests

5 cells of the putative killer strains (299 or USBL-36I1) and 5 cells of putative sensitive *Paramecium* strains (see Supplementary Table S1 online) were put together in a depression slide containing 50 μL of sterile Cerophyl or lettuce medium. Numbers of motile cells were checked after 30 and 60 minutes. 10 cells of putative sensitives were employed as controls in each experiment, which was independently repeated three times. Attempts with sterile water instead of medium and/or extended observation periods were also performed.

## Author Contributions

V.B., M.S., K.A.B., S.K., F.S., M.S. and E.V.S. participated in experimental procedures and data analysis. V.B. wrote the main manuscript text. T.U.B., M.S., F.V., E.V.S. and G.P. coordinated the work. All authors reviewed the manuscript.

## Supplementary Material

Supplementary Information Supplementary Methods
