# Supplementary material for: Rediscovering the genus Lyticum, multiflagellated symbionts of the order Rickettsiales
Source: Sci Rep. 2013 Nov 22;3:3305. doi: 10.1038/srep03305 (PMC3837311; doi:10.1038/srep03305)
Supplement: Supplementary Information — Supplementary Methods [file srep03305-s1.docx]

**Rediscovering the genus *Lyticum*, multiflagellated symbionts of the order *Rickettsiales***

Vittorio Boscaro^a†^, Martina Schrallhammer^b†^, Konstantin A. Benken^c^, Sascha Krenek^b^, Franziska Szokoli^b^, Thomas U. Berendonk^b^, Michael Schweikert^d^, Franco Verni^a^, Elena V. Sabaneyeva^c^, Giulio Petroni^a*^

^a^ Biology Department, University of Pisa

^b^ Institute of Hydrobiology, Technische Universität Dresden

^c^ Department of Cytology and Histology, St.-Petersburg State University

^d^ Department of Zoology, Biological Institute, Stuttgart University

^†^ These authors contributed equally to the paper

^*^ Corresponding author:

Giulio Petroni

Address: Biology Department, University of Pisa, via A. Volta 4/6, IT-56126 Pisa, Italy

Telephone: +39 0502211384 Fax: +39 0502211393

E-mail address: gpetroni@biologia.unipi.it

**Supplementary Methods – 16S rRNA gene sequencing**

16S rRNA gene sequencing of *Lyticum flagellatum*

The almost complete 16S rRNA gene sequence of the type strain of *L. flagellatum* (endosymbiont of *P. octaurelia* 299) was obtained through two touchdown PCRs (annealing temperature: 60 °C, 5 cycles; 58 °C, 10 cycles; 55 °C, 20 cycles; employing the high-fidelity TaKaRa Ex *Taq* and its buffer mix (TaKaRa Bio Inc., Otsu)) and direct sequencing of the amplified products. The amplification primers were: 16S F35 OdyHolo (5’-GCTGGCGGCATGCTTAAC-3’) and 1492R (5’-GGNWACCTTGTTACGACTT-3’, modified from [61]) in the first PCR; 16S Lyti F832 (5’-AGGTGTTAAAATTTTTTTAGTGCC-3’) and 16S R1522a (5’-GGAGGTGATCCAGCCGCA-3’, modified from [12]) in the second PCR. The amplified products were sequenced with the internal primers 16S R785 ND (5’-TACCAGGGTATCTAATCC-3’, [20]), 16S F515 ND (5’-GTGCCAGCAGCCGCGGT-3’, [20]), 16S F49 AlphaSym (5’-TAACACATGCAAGTCGAAC-3’) (first amplicon) and 16S Alfa R1517 (5’-TGATCCAGCCGCAGGTTC-3’, [27]) (second amplicon). The resulting sequences were compared and assembled.

16S rRNA gene sequencing of *Lyticum sinuosum*

The almost complete 16S rRNA genes of the type strain (endosymbiont of *P. biaurelia* 114) and the recently sampled strain (endosymbiont of *P. biaurelia* USBL-36I1) of *L. sinuosum* were PCR amplified using the universal primers Bac16SFor (5'-AAGAGTTTGATCCTGGCTC-3') and Bac16SRev (5'-TACGGCTACCTTGTTACGAC-3', [62]). Each reaction mix contained 1x PCR buffer (Colorless GoTaq Reaction Buffer, Promega) with 200 µM dNTPs, 2.5 mM MgCl_2_, 1 U *Taq*-polymerase (GoTaq, Promega) and 1 U DNase I (RQ1 DNase, Promega) in a total volume of 20.5 µL. This mixture was incubated for 30 min at 37 °C to degrade any containing bacterial DNA. Afterwards, 1 μL of RQ1 DNase Stop Solution (20 mM EGTA, Promega) was added and the mixture was incubated for another 15 min at 65 °C to inactivate the DNase. In a second step, 10 pmol of each primer and 2.5 µL of Chelex^®^ extracted DNA was added to reach a final volume of 25 µL. The PCRs were performed under touchdown conditions in which a 5 min initial denaturation at 95 °C was followed by 35 cycles of denaturation at 95 °C for 45 s, annealing at 63-55 °C for 45 s and extension at 72 °C for 90 s; the initial annealing temperature of 63 °C was decreased by 0.7 K at each of the first 10 cycles to reach 55 °C for the final 25 cycles. A final extension step at 72 °C for 5 min was added to facilitate ligation of PCR products into the pGEM^®^-T Vector (Promega).

PCR products were analyzed by electrophoresis on 1% agarose gel (SeaKem® LE Agarose, Lonza) stained with GelRed^TM^ (Biotium). Fragments of the right size (approx. 1.5 kb) were excised, purified with the NucleoSpin^TM^ Extract II kit (Macherey-Nagel) and subsequently cloned and transformed into competent *E. coli* JM109 cells using the pGEM®-T Vector System (Promega) and following the manufacturer’s instructions.

Picked colonies from agar plates were resuspended into 20 µL ddH_2_O and incubated for 15 min at 95 °C for cell lysis. 10 µL of this suspension was used for colony PCR with the following components: 1x PCR buffer (Colorless GoTaq Reaction Buffer, Promega) with 50 µM dNTPs, 2 mM MgCl_2_, 1 U *Taq*-polymerase (GoTaq, Promega) and 5 pmol of both M13 forward and reverse primer in a total volume of 20 µL. PCR conditions were: 3 min initial denaturation at 94 °C, followed by 35 cycles of 30 s at 94 °C, 30 s at 60 °C, 90 s at 72 °C and a final extension step of 5 min at 72 °C.

To identify colonies containing the 16S rRNA gene fragments of interest (derived from endosymbionts and not bacterial contaminants), PCR products were digested with the restriction enzyme MboI (Promega). Five clones derived from DNA of either strain 114 or USBL-36I1 showing the same and most represented restriction pattern were sequenced from both directions using standard M13 primers. The resulting five sequences of each strain were used to produce a consensus sequence for each of the two endosymbionts.

Additional references not included in the main text:

1. Lane, D. J. [16S/23S rRNA sequencing] *Nucleic Acid Techniques In Bacterial Systematic* [Stackebrandt, E. & Goodfellow, M. (eds.)] (Wiley, New York, 1991).
2. Neilan, B. A. et al. rRNA sequences and evolutionary relationships among toxic and nontoxic cyanobacteria of the genus *Microcystis*. *Int. J. Syst. Bacteriol.* **47**, 693-697 (1997).

**Supplementary Table 1 – strains employed during killer tests**

| **Putative sensitive strain** | **Species** |
| --- | --- |
| 562 | *Paramecium biaurelia* |
| 325 | *Paramecium triaurelia* |
| Alpha4-2 | *Paramecium tetraurelia* |
| Hp1-9 | *Paramecium pentaurelia* |
| 159 | *Paramecium sexaurelia* |
| GFG-1 | *Paramecium octaurelia* |
| 223 | *Paramecium decaurelia* |
| UV | *Paramecium dodecaurelia* |
| Rs12 | *Paramecium dodecauarelia* |
| 209 | *Paramecium tredecaurelia* |
